# Impact of SARS-CoV-2/COVID-19 on Provision of Medical Care to Patients With Systemic Autoimmune Rheumatic Disease and the Practice of Rheumatology

**DOI:** 10.7759/cureus.35402

**Published:** 2023-02-24

**Authors:** Adegbenga A Bankole, Jane Nwaonu, Jahanzeb Saeed

**Affiliations:** 1 Rheumatology, Virginia Tech Carilion School of Medicine, Roanoke, USA; 2 Internal Medicine, Virginia Tech Carilion School of Medicine, Roanoke, USA; 3 Internal Medicine, Carilion Clinic, Roanoke, USA

**Keywords:** autoimmune rheumatic disease, myalgic encephalomyeritis/chronic fatigue syndrome, vaccine science and policy, cytokine strom, immunosuppressive therapies, covid long haul syndrome, autoantibody, health policy and economics, telemedicine (tm), covid-19

## Abstract

The SARS-CoV-2 pandemic has had a significant impact on the healthcare field that resulted in changes to the way safe and effective medical care is delivered. The effects range from service disruption including ambulatory clinic closure due to both patient and provider concerns, to lack of capacity in hospital services. In rheumatology, there were other effects including viral infection-related autoantibody production, concerns about the use of systemic immunosuppression in the presence of an infectious pandemic and even concerns for viral infection-induced flares of rheumatic disease.

Coronavirus disease 2019 (COVID-19) led to the rapid adoption of innovative technologies that permitted the introduction and increased use of telemedicine via a number of platforms. Rapid discoveries and innovations led to the development of diagnostic and therapeutic agents in the management of COVID-19. Scientific advancement and discoveries around COVID-19 infection, symptoms, autoantibody production, chronic sequela and the repurposing of rheumatic immunosuppressive agents led to improved survival and an expanded role for the rheumatologist.

Rheumatologists may sometimes be involved in the diagnosis and management of the hospitalized COVID-19 patient. In the ambulatory clinic, a rheumatologist also helps to differentiate between symptoms of long COVID and those of systemic autoimmune rheumatic disease (SARD). Rheumatologists must also grapple with the concerns related to immunosuppressive therapy and the risk of COVID-19 infections. In addition, there are concerns around vaccine effectiveness in people with SARD and those on immunosuppressive medications. Although the SARS-CoV-2 pandemic and the effects on healthcare resulted in difficulties, both patients and providers have risen to the challenge. The long-term outcome of COVID-19 for the medical system and rheumatologists in particular is not yet fully understood and will need further study. This review concentrates on the changing role of the rheumatologists, improved understanding of rheumatic disease and immunosuppressive therapies in the wake of the pandemic and how this has led to an improvement in the care of patients with COVID-19.

## Introduction and background

The first cases of coronavirus disease 2019 (COVID-19) caused by severe acute respiratory syndrome coronavirus 2 (SARS-CoV-2) were reported in 2019 in Wuhan, China. By February 2020, COVID-19 had spread around the world [1]. At that time, there was no recognized pharmacological treatment for COVID-19, and the world focused on the interruption and prevention of the community-hospital-community transmission cycle and human-to-human transmission outside the hospital via enhanced traffic control bundling, social distancing, improved hand hygiene, and masking [2,3]. With the increasing number of cases around the world, the Centers for Disease Control and Prevention (CDC), taking cues from other health agencies, imposed travel restrictions for those planning to travel to the USA [4]. Despite these efforts, the number of cases continued to increase and as of March 2022, there had been approximately 80 million cases with over 960,000 deaths attributed to COVID-19 in the USA alone [5]. The steps needed to combat COVID-19 and reduce healthcare-associated spread resulted in the reduction of engagement with the healthcare team, and reduced utilization of emergency and non-emergency medical care [6,7]. In some cases, such delays were associated with excess morbidity and mortality from both SARS-CoV-2 and non-SARS-CoV-2 disease [8]. Due to the potential risk of spread, recommendations from multiple agencies included a reduction in outpatient encounters, especially in patients with systemic autoimmune rheumatic disease (SARD) [9].

This pandemic also adversely impacted the health sector, including rheumatology. It affected staff morale, and the reduction in demand for elective, acute hospital services, and ambulatory services also had a financial impact [10]. The increase in unemployment led to a loss of employer-based health insurance. The loss of health insurance and the cost of care of patients with COVID-19 had financial implications for patients, healthcare providers and healthcare systems.

Although there was a major decline in the number of patient visits, the medical system adjusted by rapidly adopting telemedicine [11]. Several barriers that had prevented the earlier utilization of telemedicine, including both regulatory and reimbursement issues, were addressed as a result of the pandemic [12]. Telemedicine allowed patients to continue to receive care safely and securely, once both the healthcare providers and patients began to understand this system [13]. Telemedicine was embraced early by rheumatologists specifically due to concerns of increased risks of developing severe COVID-19 infection and the outcomes in patients on immunosuppressive medications. The move to telemedicine was not just regional, as rheumatologists around the world had the same concerns and initiated similar solutions [14].

Given the varied impact of this infection on healthcare and the particular impact on the discipline of rheumatology, the goal of this article is to discuss COVID-19 and the particular ways it influenced rheumatology.

## Review

COVID-19 symptoms and the role of rheumatologists

**Figure 1:**
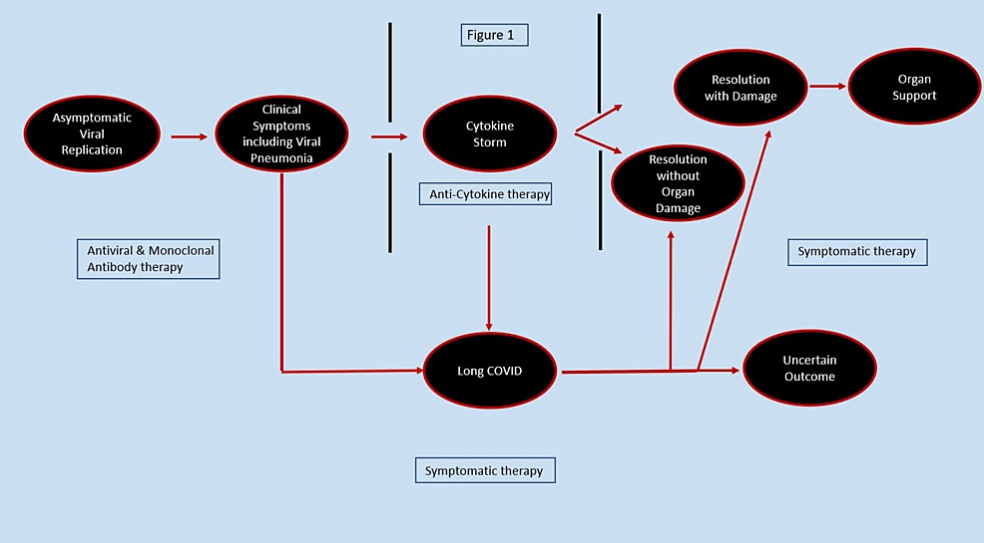
Clinical symptoms, therapeutics, natural history and clinical outcome

The SARS-CoV-2 virus is highly transmissible, spreading person to person via infected droplets, contact with moist mucosal surfaces, and contaminated surfaces [15]. The clinical symptoms of COVID-19 range from asymptomatic infections, with the majority of people having what is described as mild to moderate disease with a small percentage of patients becoming critically ill (Figure 1).

The manifestations of COVID-19 involve multiple organs including the respiratory, neurological, gastrointestinal, renal, cardiac, and vascular systems [15]. Some patients also develop lingering symptoms following viral clearance that persist after the initial acute viral infection that many refer to as ‘long COVID’, ‘long haulers syndrome’ or ‘post-COVID syndrome’ [16]. Some patients also develop clinical findings that are difficult to separate from new-onset SARD [17].

Some authors classify COVID-19 symptoms and complications into two major categories, i.e., inflammatory and post-inflammatory symptoms. In addition to the typical respiratory findings, some other inflammatory symptoms of COVID are similar to symptoms of SARD, and include arthralgia, inflammatory arthritis, glomerulonephritis, vasculitis, myalgias, and inflammatory muscle disease. In addition, severe symptoms are related to cytokine storm that are potentially serious and need prompt intervention. It can be difficult to determine if these inflammatory symptoms are an exacerbation of underlying SARD, de novo development of SARD following COVID-19, or purely symptoms of COVID-19 [18].

COVID-19 can result in organ damage that results in lingering symptoms. Some neuropsychiatric symptoms also form part of the post-inflammatory sequelae of COVID-19. This collection of symptoms is now known as long COVID. The National Institute for Health and Care Excellence (NICE) defines long COVID as signs and symptoms that continue or develop after acute COVID-19. It includes both ongoing symptomatic COVID-19 (between 4 and 12 weeks) and post-COVID-19 syndrome, with symptoms presenting for 12 weeks or longer [19]. The neuropsychiatric symptoms of long COVID are also varied and may include fatigue, diffuse muscle pain, weakness, depression, and sleep disturbance [20]. These symptoms are consistent with symptoms of fibromyalgia, post-exertional malaise (PEM) and myalgic encephalomyelitis (ME) and may be confused with symptoms of SARD [21-24]. As it can be difficult to differentiate these neuropsychiatric symptoms from exacerbations of pre-existing neuropsychiatric disease including neuropsychiatric systemic lupus erythematosus (NPSLE) and fibromyalgia, rheumatologists can sometimes be helpful in the care of these patients. There is currently no agreed-upon management plan for long COVID, but given the overlap in symptoms, the management is similar to PEM.

COVID-19, autoantibodies, cytokines and rheumatology

Similar to a number of other viral infections, patients with moderate-to-severe COVID-19 infections (including hospitalized patients) may develop novel autoantibodies via numerous mechanisms including molecular mimicry, expression of modified, cryptic, or novel antigenic determinants, superantigen infections, and bystander activation [25-27]. The vast majority of autoantibodies occur in moderate-to-severe disease where hospitalization is needed (Table 1). The clinical significance of these autoantibodies is not always obvious, as they are not always associated with the development of SARD [28]. Despite the reported symptoms ranging from arthralgia to severe vasculitis, some of which is suggestive of SARD, patients do not always develop or meet the diagnostic or clinical criteria for SARD [29,30]. Given the incidence of autoantibodies in COVID-19, and that autoantibodies tend to occur prior to the development of SARD (pre-clinical autoimmunity), it is not unreasonable to expect an increase in the incidence of SARD following the COVID-19 pandemic. In addition to the risk of the development of SARD, there is also concern that there could be a significant increase in flares of anti-nuclear antibody (ANA)-mediated diseases including SLE.

**Table 1:** Autoantibody production in patients admitted with COVID-19 anti-SSA: anti-Sjogren's syndrome A; anti-SSB: anti-Sjogren's syndrome B; anti-CCP: cyclic citrullinated peptide antibody; anti-TPO: anti-thyroid peroxidase; ANCA: anti-neutrophil cytoplasmic antibody (C, cytoplasmic; P, perinuclear)

Autoantibody production in patients admitted with COVID-19					
Item	Incidence rate (%)	Number of patients	Control group	Study design	Reference
Anti-nuclear antibody	33	33	Yes	Prospective	Pascolini et al. [31]
Anticardiolipin antibody	27.4	157	No	Retrospective	Bertin et al. [32]
Anti-SSA antibody	50	21	No	Retrospective	Zhou et al. [33]
Anti-SSB antibody	29	7	No	Retrospective	Gracia-Ramos et al. [34]
Antiphospholipid antibody	57	21	No	Case series	Amezcua-Guerra et al. [35]
Anti-smooth muscle antibody	7.4	84	No	Retrospective	Richter et al. [36]
Epidermal (intracellular) antibody	10	84	No	Retrospective	Richter et al. [36]
Type I interferon antibody	10	3,589	No	Retrospective	Bastard et al. [37]
Lupus anticoagulant	11	45	No	Retrospective	Gazzaruso et al. [38]
B2-glycoprotein	9	32	No	Retrospective	Liu et al. [18]
Rheumatoid factor	19	21	No	Retrospective	Gracia-Ramos et al. [34]
Anti-CCP antibody	24	68	No	Retrospective	Lingel et al. [39]
Anti-TPO antibody	8	147	Yes	Retrospective	Chang et al. [17]
Anti-globulin antibodies	44	113	Yes	Retrospective	Berzuini et al. [40]
ANCA	25	40	No	Retrospective	Sacchi et al. [41]
C-ANCA/P-ANCA	72/28	100	No	Retrospective	Kadkhoda et al. [42]

**Figure 2:**
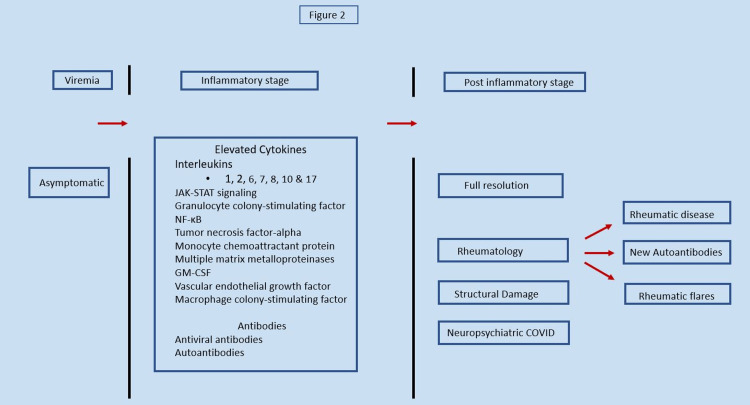
Cytokines associated with clinical symptoms JAK-STAT: Janus kinase/signal transducer and activator of transcription; NF-κB: nuclear factor kappa B; STAT3: signal transducer and activator of transcription 3; GM-CSF: granulocyte–macrophage colony-stimulating factor

In addition to antibody production, COVID-19 also causes marked inflammation. This inflammation is related to the very high levels of cytokines (aka hypercytokinemia or cytokine storm) that can cause significant mortality and morbidity. The clinical symptoms of COVID-19 relate to several cytokines including interleukin (IL)-2, IL-7, IL-10, tumor necrosis factor (TNF) with IL-6, IL-6-signal transducer and activator of transcription 3 (STAT3) and nuclear factor kappa B (NF-κB) pathway being of high significance (Figure 2) [43]. Multisystem inflammatory syndrome in children (MIS-C) is a Kawasaki disease-like cytokine syndrome widely reported in children with COVID-19; this hyper-inflammatory syndrome affects multiple organs presenting two to four weeks following infection [44].

Since coagulopathies and other related findings were first reported as a feature of COVID-19, it has become recognized as a major source of morbidity and mortality [45]. Although multifactorial in nature, there is a strong immune-mediated component with respect to the development of antiphospholipid antibodies that are functional and lead to thrombosis [26].

Non-rheumatic immune-mediated diseases have also been reported in COVID, including immune-mediated neuropathic, hematologic, and endocrine disorders.

COVID-19, telemedicine and rheumatology

Prior to the current pandemic, the utilization of telemedicine was low in the rheumatology field given the complex nature of SARD and the success of telemedicine/telerheumatology was considered uncertain in the management of these complicated diseases [46]. In the first few weeks of the pandemic, healthcare providers (including rheumatologists) and many patients on immunosuppressive medications shared the common concern that this group of patients would be at higher risk of contracting COVID-19, having more severe disease, and poorer outcomes. This concern generated interest in alternative ways of conducting patient visits. Concerns around virtual medicine were related to the availability of the required equipment, barriers to the utilization of the technology and the inability to perform some elements of the physical examination. These concerns were more acute in new patients given the importance of establishing an accurate diagnosis. Given the importance of the act of physical touch in expressing empathy, building trust, and developing the relationship between providers and patients, it was unclear how the lack of physical contact especially in new patients would impact the patient-provider relationship [47]. In academic health centers, there was also a concern surrounding medical training and education and gaps in the learning of students and residents [48].

Despite these initial concerns, more recent studies have demonstrated that even though physical contact and physical examination were missed by patients and providers alike, the vast majority of people were satisfied with telemedicine as a method of providing care during the current pandemic. The majority of patients and healthcare providers showed a desire to continue telemedicine in some form post-pandemic. The exact role of telemedicine when managing new patient visits and complicated patients with SARD has not been fully determined, as these patients may benefit from an in-person visit for a comprehensive assessment [49]. Some studies suggest that a significant number of patients with SARD prefer telerheumatology, finding it more convenient and helping to alleviate concerns around COVID-19 [50]. There are certain situations where both patients and providers prefer an in-person visit, including when interpreters are required, especially when it is related to discussions about treatment [51]. In order of preference, in-person visits were ranked higher, with video and telephone visits being the least liked prior to the pandemic [51]. The general experience with virtual visits during the pandemic has made us all more open to the necessity of change.

COVID-19, immunosuppression and immunosuppressive agents

There was a concern during early days of the pandemic among rheumatologists that there would be a considerable risk of contracting and developing severe COVID-19 in patients with or being treated for SARD [52]. In addition, there were concerns that lowering the dose or withdrawing immunosuppressive therapies could lead to an increase in the risk of flares leading to undesired outcomes [53]. Concerns around COVID-19 within the rheumatology community led to the rapid development of the COVID-19 Global Rheumatology Alliance allowing for these topics to be scientifically studied. The development of this research group was instrumental in exploring the effects of COVID-19 on rheumatology patients and the community. One of their early reports confirmed an increased risk of hospitalization of patients with SARD and COVID-19 on high-dose glucocorticoids (GCs) and a decreased hospitalization risk with TNF inhibitor use [54]. Furthermore, a meta-analysis of over 10,000 patients performed in 2021 showed that compared to the general population, people on immunosuppressive medications were not at a significantly higher risk of COVID-9 [55].

Large observational studies confirmed that in general, the risk factors associated with adverse outcomes in patients with SARD are similar to the general population [56]. However, combination therapy that is commonly used to manage severe rheumatoid arthritis (RA) and lupus nephritis including high-dose GCs and cyclophosphamide in patients over 50 years is associated with a higher risk of COVID-19 infection, hospitalization and poorer outcomes [57,58].

Some biological disease-modifying anti-rheumatic drugs (DMARDs) such as TNF-α inhibitors may reduce the risk of COVID-19 [59]. Along with low-dose prednisone, TNF-α inhibitors are associated with a lower rate of COVID-19 hospitalization and improved outcomes [54,60]. In general, neither a diagnosis of SARD nor the use of DMARDs has been conclusively associated with a higher risk of COVID-19 [55,61]. The early concerns however led to therapeutic changes, including skipping, reducing or stopping medications, even in those with disease activity and severe disease [62,63]. The current understanding of what clinically constitutes a high-risk COVID-19 patient and the interplay of SARD and immunosuppressive therapies in COVID-19 has resulted in modifications to the care of these patients. To guide providers, a number of national rheumatology associations have consensus statements about the use of DMARDs during the COVID-19 era.

The understanding of the role of cytokines in COVID-19 and the related cytokine storm also led to the development of evidence-based treatment algorithms that included many agents familiar to the rheumatologists. The use of rheumatic-based immunosuppressives targeting hypercytokinemia in patients with COVID-19 cytokine storm has put rheumatologists on the frontline of care in the critically ill COVID-19 patients in many centers. Rheumatologists now have to balance decisions on immunosuppressive therapy for patients in need of respiratory and cardiovascular support because of a severe viral infection, while still providing care to those with SARD and confirming that immunosuppressive therapy does not necessarily lead to higher risk of COVID-19 [52,55,60,61,64].

COVID-19 and disease-modifying agents

At the onset of the pandemic, the high morbidity and mortality meant that there was an urgent need for therapeutics. The length of time needed for the development of new agents meant that the initial focus was on repurposing existing antiviral agents including those already approved or in development for Ebola, Middle East respiratory syndrome coronavirus (MERS-CoV), HIV, hepatitis B virus (HBV), hepatitis C virus (HCV), and influenza [59,65]. Remdesivir, a nucleotide analog, was the first direct-acting antiviral to receive FDA Emergency Use Authorization (EUA) in 2020 for the treatment of COVID-19 patients. EUA for the emergency use of molnupiravir and nirmatrelvir-ritonavir (Paxlovid) followed in 2021 [66].

The mechanism of action of chloroquine (CLQ) and hydroxychloroquine (HCQ) led to them being considered as potential therapeutic agents [67]. Due to the improved safety profile, HCQ was already widely used in rheumatology, especially in diseases like SLE, and repurposing HCQ appeared to be a logical step in the care of COVID-19 patients [68]. Many meta-analyses confirmed that this group was not effective in the prevention or treatment, and the medical community moved on quickly [67,69].

Although none of the DMARDs used in rheumatology have shown efficacy as an anti-viral or in the prevention of SARS-CoV-2 infection, they have a role in the treatment of SARS-CoV-2-related hyperinflammation and cytokine storm. Early investigations showed several cytokines at increased levels (hypercytokinemia) in COVID-19 and the related cytokine storm [70]. The hypercytokinemia and the resultant inflammatory response was related to the severity of infection and outcomes in patients, and this association portends further biological plausibility for these agents in the treatment of COVID-19 [71]. Some of these cytokines had commercially available agents targeting them that rheumatologists were already familiar with. One of the most commonly targeted cytokines in rheumatology was TNF alpha, and as the use of TNF inhibition in patients with SARD was associated with less frequent COVID-19 infection, it was natural for this to be a potential treatment for COVID-19 [72]. However, the inhibition of TNF alpha was not found to be helpful [73]. IL-6, IL-6-STAT3, and the NF-κB signaling pathway are markedly activated in hypercytokinemia associated with COVID-19 [55]. IL-6 levels are also a marker for the development of COVID-19-related pneumonia and respiratory failure, and a prognosticator for the survival rate. Multiple studies including meta-analyses have confirmed the relationship between COVID-19, disease severity, and IL-6 levels [74]. Early studies confirmed that inhibiting IL-6 was a useful target in hospitalized patients with COVID-19-related pneumonia especially in the highest-risk group of patients [75]. In these early studies, there was no mortality benefit, but the studies confirmed significant improvement in the requirement for respiratory support with mechanical ventilation [76]. Tocilizumab (TCZ) and sarilumab, both of which are monoclonal antibodies that block IL-6, showed benefits in COVID-19 when given early [77]. They demonstrated a significant reduction in mortality in the highest risk patients including those with a high C-reactive protein (CRP) level, and in the elderly where outcomes are worse [78]. Despite this and the fact that it quickly resolves clinical manifestations including fever and oxygen saturation, there is still controversy around it as a phase 3 randomized trial of hospitalized patients with severe COVID-19 pneumonia did not show benefit in clinical status and/or mortality rates at 28 days [79].

IL-1, an important cytokine involved with the inflammasome activation and the inflammatory response, is also involved in COVID-19 [80]. Inhibition of this pathway is most often used by rheumatologists in the management of periodic fever syndromes that have a number of symptoms similar to the cytokine storm seen in COVID-19 [81]. IL-1 inhibition does show promise in COVID-19 [82]. Although there is an interplay between IL-1, IL-6 and multiple other cytokines, IL-6 inhibition appears to be superior to IL-1 inhibition in COVID-19 and remains the main focus of therapy [83].

GCs commonly form an important part in the first-line treatment in controlling the clinical symptoms of SARD, as well as in critical conditions such as septic shock [84]. GCs are effective in treating SARD with high cytokine levels as well as in hypercytokinemia caused by infectious agents. Given this, GCs were investigated as a potential therapeutic agent in COVID-19. Both low and high doses of GCs have shown benefits in COVID-19 hospitalized patients. Dexamethasone is the preferred agent, and it has shown a reduction in all-cause mortality at 28 days [85]. The use of GCs has become the standard of care in COVID-19 and has led to improvement in outcomes in patients as noted in a large meta-analysis [86]. In appropriate patients, the analysis suggested that combining dexamethasone with TCZ confirmed a synergistic effect when given within the first 10 days [87]. There are ongoing clinical trials comparing tocilizumab and dexamethasone versus dexamethasone in patients with COVID-19. SARS-CoV-2 infects cells through endocytosis via the adaptor-associated protein kinase 1 (AAK1) pathways that utilizes the Janus kinase (JAK) 1 and 2 pathways. JAK1 and JAK2 pathways are also involved in pathways that result in COVID-19 hypercytokinemia; therefore, therapeutic agents that target these pathways may have a role in the treatment of COVID-19 [88]. Ruxolitinib is a selective JAK1 and JAK2 inhibitor that also has modest selectivity against tyrosine kinase (TYK) 2 and JAK3 and shows benefit in COVID-19 [89]. Baricitinib is a selective JAK1/JAK2 inhibitor approved for the treatment of RA and has shown significant morbidity and mortality benefits in COVID-19 [90]. The Janus kinase signaling pathway was also identified as a potential contributor to the thrombotic and cytokine storm-related symptoms. The concern with JAK inhibitors is the relationship between these agents and thrombosis risk in post-marketing data in diseases like RA [91].

While some agents have shown benefits, other medications have been shown to worsen outcomes. The use of rituximab (RIX), an agent that depletes B cells, has shown an increase in the need for mechanical ventilation and in-hospital deaths, confirming older descriptive findings [92,93].

COVID-19 and vaccination

With the rollout of COVID-19 vaccinations, there were initial concerns from both patients and providers about the possible reduced humoral response to these vaccines. Studies have shown that there is an adequate response in most patients with SARD on immunosuppressive therapies [81]. Given that the humoral response is of clinical significance in protecting these patients against COVID-19, there is continued concern about the lower seroconversion rates in patients on RIX therapy [82]. Several studies have confirmed that pre-treatment with RIX reduces the rate of vaccine response, and the timing of RIX treatment is the key factor [94]. A similar concern is noted in patients on mycophenolate, and analysis of data on SLE patients who tend to be treated with higher doses of mycophenolate reported reduced rates of vaccine protection [95]. Overall, outside of patients using high doses of steroids, RIX and mycophenolate, these vaccines produce a good response and are effective in reducing the rates of COVID-19 in this group of patients [96]. Booster vaccinations further increase vaccine seroconversion, but this response is still impaired in patients on high doses of steroids, RIX and mycophenolate [97].

## Conclusions

In rheumatology, we have had to contend with some unique consequences of COVID-19, including the production of autoantibodies, and development, exacerbation, or emergence of SARD. Although there are gaps in our knowledge regarding COVID-19 and the related long-term complications, we continue to make great strides in the treatment of this disease. Rheumatologists will need to continue monitoring patients with SARD who develop COVID-19. We will need to be actively involved in the medication management in order to prevent changes that may result in disease flare. There will also be a role for rheumatologists in monitoring patients who develop COVID-19-induced autoantibodies, as disease surveillance and early intervention may be needed to prevent complications from new-onset SARD.

Given all of the current unknowns, COVID-19 and its study remain an active field of research. Medical care providers including rheumatologists will need to remain well informed of developments of this infectious disease. As a discipline, rheumatology adapted well to virtual medicine. We all should remain open to other changes that may help improve the way we provide care in the future. This is important as this current pandemic resulted in changes in patient expectations.
